# Epidemiology and outcomes of elderly patients requiring renal replacement therapy in the intensive care unit: an observational study

**DOI:** 10.1186/s12882-021-02302-4

**Published:** 2021-03-19

**Authors:** Cécile Salathé, Elettra Poli, Marco Altarelli, Nathan Axel Bianchi, Antoine Guillaume Schneider

**Affiliations:** 1 Adult Intensive Care Unit, Service de Médecine Intensive Adulte, Centre Hospitalier Universitaire Vaudois (CHUV), 46, Avenue du Bugnon, 1011 Lausanne, Switzerland; 2 Intensive Care Unit, Réseau Hospitalier Neuchâtelois (RHNE), Neuchâtel, Switzerland; 3 Faculty of Biology and Medicine, University of Lausanne, Lausanne, Switzerland

**Keywords:** Renal replacement therapy (RRT), Acute kidney injury (AKI), Elderly, Mortality, Quality of life (QOL)

## Abstract

**Background:**

Renal replacement therapy (RRT) in critically ill patients is associated with high morbidity and mortality. The appropriateness of RRT initiation is sometimes questioned in elderly patients. Therefore, we sought to evaluate the long-term mortality, dialysis dependence and quality of life (QOL) of elderly patients who survived critical illness requiring RRT.

**Methods:**

This is a monocentric observational study including all patients > 55 yo who received RRT for acute kidney injury in our intensive care unit (ICU) between January 2015 and April 2018. At the time of the study (May 2019), we assessed if they were still alive by cross referencing our hospital database and the Swiss national death registry. We sent survivors written information and, subsequently, contacted them over the phone. We obtained their consent for participation, asked about their dialytic status and performed an EQ-5D survey with visual analog scale (VAS). Results were stratified according to their age at the time of ICU admission (G1: “55–65 yo”; G2: “> 65–75 yo” and G3: “> 75 yo”). QOL in G3 patients were compared to G1 and G2 and to predicted values.

**Results:**

Among the 352 eligible patients, 171 died during the index hospital admission. After a median follow-up time of 32.7 months (IQR 19.8), a further 62 had died (median time to death for ICU survivors 5.0 (IQR 15.0) months. Hence, 119 (33.6%) patients were still alive at the time of the study. We successfully contacted 96 (80.7%) of them and 83 (69.7%) were included in the study (G1: 24, G2: 44 and G3: 15). Only 6 (7.2%) were RRT dependent. Patients in G3 had lower EQ-5D and VAS scores than those in G1 and G2 (*p* < 0.01). These scores were also significantly lower than predicted values (*p* < 0.05).

**Conclusions:**

RRT patients have a very high in-hospital and post discharge mortality. Among survivors, RRT dependency was low. Irrespective of baseline values, patients > 75 yo who survived ICU had a lower QOL than younger patients. It was lower than predicted according to age and sex. The appropriateness of RRT initiation in elderly patients should be discussed according to their pre-existing QOL and frailty.

**Supplementary Information:**

The online version contains supplementary material available at 10.1186/s12882-021-02302-4.

## Background

There is growing interest in long-term outcomes of elderly patients admitted to the intensive care unit (ICU). Indeed, together with populations aging and increasing life expectancy, the admission rate of elderly patients is increasing in modern ICUs. However, such admissions are associated with a very high in-hospital mortality, approaching 50% in patients over 80 years old [1, 2]. Hence the appropriateness of their admission to ICU is regularly questioned.

Beyond mortality, quality of life (QOL) appears to be a key element in determining quality of care in elderly patients. 80% of > 80 yo patients, appeared to be self-sufficient for daily activities 12 months after discharge from ICU [2]. Other studies confirmed fair QOL in those patients, for instance 75% of those who lived at home on admission, were still living at home 1 year after discharge [3]. In general, most authors consider that age, even > 90 years, should not withhold ICU admission provided pre-admission QOL is satisfactory [4].

However, large uncertainty remains in elderly patients with severe AKI and RRT. Indeed, irrespective of age, AKI is a frequent complication in the ICU and the need for RRT is associated with a very high mortality. Among those who survive, a large number will develop chronic kidney disease [5, 6]. However, their QOL 6 months after discharge, was evaluated to be comparable with the QOL of similar patients who did not require RRT [7].

Little is known about long-term outcomes of elderly patients admitted to the ICU who underwent RRT for AKI. Cohort studies have confirmed that, in patients receiving RRT, age was associated with a very high mortality (> 70% in > 65 yo patients) [8, 9]. However, data suggest that their length of stay (LOS) or chronic dialysis at hospital discharge was similar to younger patients [10]. Again, authors concluded that age alone should not withhold therapeutic measures, especially dialysis [11], however data supporting this statement remain scarce.

We hypothesized that elderly patients surviving ICU after RRT might have higher mortality and lower QOL than their younger counterparts. Based on this hypothesis, we designed an observational study aiming at evaluating long-term mortality and QOL of elderly patients who require RRT while in ICU.

## Methods

### Study design

This is a monocentric observational study conducted in a tertiary teaching hospital located in Lausanne, Switzerland. The ICU contains 35 beds and records approximately 2000 admissions per year. All consecutive ≥55 yo patients who received RRT for AKI in our ICU between 1.1.2015 and 30.04.2018 were included in the study. For all patients, RRT was provided in the form of continuous RRT and AKI was defined according to Kidney Disease Improving Global Outcomes (KDIGO) criteria. Patients with end-stage kidney failure on chronic dialysis and those who declined institutional consent for data reutilization, were excluded.

We assessed if the included patients were still alive at the time of the study (May 2019), by cross referencing our hospital database and the Swiss national death registry 12 months after the end of the study period. Mortality and time to death were recorded. Patients still alive underwent a phone interview, during which they were asked about their dialytic status. Their QOL was evaluated using an EQ-5D-3L survey with visual analog scale (VAS).

### Ethics

The study protocol was approved by the Ethics Committee Vaud (CER-VD 2019–00359). In our institution, consent for data reutilization is sought for each patient and those who declined such consent were excluded from the study (irrespective of their vital status at the time of the study).

Eligible survivors were sent a written description of the study by regular mail. Approximately 10 days later, they were contacted by phone in order to obtain consent and collect responses for the survey. The need for informed consent was waived for eligible patients who were deceased at the time of the study.

### Data collection

#### Patients’ characteristics and outcomes

All data were collected using electronic chart records (Metavision®, IMD Soft, Tel Aviv, Israel) and Soarian® (Cerner, North Kansas City, USA). We collected patient’s characteristics on admission as well as ICU and hospital outcomes (survival, length of stay and dialysis dependence). For patients with multiple ICU admissions, only the first was considered for baseline data and the last for outcome data.

Study data were collected and managed using REDCap electronic data capture tools hosted in our institution [12].

#### Health utility instrument (EQ-5D-3L)

The EQ-5D 3 L [13, 14] is a standardized health-related QOL questionnaire which is simple to apply, validated in several languages including French [15] and convenient to use over the phone with elderly people [16]. Participants must score five items: mobility, self-care, daily activities, pain/discomfort and anxiety/depression from 1 (no problem) to 3 (serious problem). The final output can be converted into a health utility score based on local population calibration. In the absence of a Swiss calibration, we have used the French calibration [https://cran.r-project.org/web/packages/eq5d/eq5d.pdf].

The EQ-5D also includes a visual analogue scale (VAS) for self-estimation of health status. Patients are asked to rate their health status between 0 (worst) and 100 (best imaginable) (Fig. S1).

### Statistical analysis

Continuous data with normal distribution are reported as mean and standard deviation and compared with t-test. Continuous data with non-normal distribution are reported as median (interquartile range) and compared with Mann-Whitney test. Categorical data are reported as number and percentage and compared with Pearson Chi-square or Fisher’s exact test. Analyses were stratified according to age groups G1: “55–65 yo”, G2: “> 65–75 yo” and G3: “> 75 yo” based on patients’ age at the time of the index admission. Comparisons were made between groups with ANOVA or Kruskall-Wallis according to data distribution. Mortality across the three subgroups was reported using Kaplan-Meier curves and compared with log rank test. Predicted health utility and VAS scores for each patient were calculated based on equations derived from French speaking Swiss reference values [15]. Such calculations account for age and gender. Observed – predicted difference was computed and compared with one sample- t test. Multivariable logistic regression analysis was applied to the assessment of possible confounders in the association between age and predicted health utility or VAS scores. Considered variables were sex, hospital length of stay, presence of any comorbidity and SAPS score. A *p* value < 0.05 was considered to be statistically significant.

## Results

### Patients’ demographics

During the study period (Fig. S2), 6′632 patients were admitted to our ICU. Of those, 608 (9.2%) received RRT. After exclusion of patients younger than 55 yo (*N* = 135), on chronic dialysis (*N* = 84), and those who declined institutional consent for data reutilization (*N* = 37), we identified 352 patients eligible to enter this study. Median length of time from ICU discharge to the survey, was 32.7 months (IQR 19.8). Patients’ characteristics on ICU admission are displayed in Table 1.

**Table 1:** Patients’ demographics

	TOTAL 352	55–65 yo 89 (25.3%)	> 65–75 yo 162 (45.8%)	> 75 yo 101 (28.7%)	***p*** value
Median age - years (IQR)	70.3 (11.3)	60.0 (4.8)	70.1 (4.9)	78.9 (5.7)	< 0.001
Gender Male - n (%)	270 (76.7%)	77 (86.5%)	115 (71.0%)	78 (77.2%)	0.02
Median weight - kg (IQR)	80.0 (20.0)	80.0 (24.0)	80.0 (20.0)	75 (17.0)	0.35
Admission year					0.16
2015	96 (27.3%)	23 (25.8%)	44 (27.2%)	29 (28.7%)	
2016	100 (28.4%)	19 (21.3%)	55 (34.0%)	26 (25.7%)	
2017	119 (33.8%)	38 (42.7%)	50 (30.9%)	31 (30.7%)	
2018	37 (10.5%)	9 (9.0%)	13 (8.0%)	15 (14.9%)	
Admission type – n (%)					0.90
Medical	152 (43.2%)	39 (43.8%)	73 (45.1%)	40 (39.6%)	
Surgical	170 (48.3%)	42 (47.2%)	15 (46.3%)	53 (52.5%)	
Other	30 (8.5%)	8 (9.0%)	14 (8.6%)	8 (7.9%)	
Median SAPS Score (IQR)	56 (17)	53 (22)	56 (13)	59 (13)	0.27
Median ICU LOS – days (IQR)	8.6 (16.0)	12.9 (18.8)	8.8 (15.3)	7.7 (14.5)	0.10
Median hospital LOS – days (IQR)	20.0 (35.7)	24.1 (45.0)	18.6 (36.3)	18.0 (31.8)	0.39
Co-existing conditions – n (%)	197 (56.0%)	53 (59.6%)	91 (56.2%)	53 (52.5%)	0.71
Chronic kidney disease	87 (25.4%)	13 (15.3%)	42 (26.4%)	32 (32.7%)	0.03
Chronic heart disease	63 (17.9%)	15 (16.9%)	31 (19.1%)	17 (16.8%)	0.83
Chronic respiratory disease	53 (15.1%)	9 (10.1%)	24 (14.8%)	20 (19.8%)	0.12
Chronic liver disease	29 (8.2%)	10 (11.2%)	12 (7.4%)	7 (6.9%)	0.50
Immunosuppression	51 (14.5%)	18 (20.2%)	24 (14.8%)	9 (8.9%)	0.09
Haematological	31 (8.8%)	10 (11.2%)	14 (8.6%)	7 (6.9%)	0.58
Metastatic cancer	28 (8.0%)	6 (6.7%)	16 (9.9%)	6 (5.9%)	0.44
On RRT initiation					
Mechanical ventilation – n (%)	269 (75.9%)	69 (77.5%)	124 (76.5%)	74 (73.3%)	0.76
Noradrenaline – n (%)	302 (85.8%)	72 (80.9%)	138 (85.2%)	92 (91.1%)	0.05
Mean max creatinine - mmol/l (SD)	314.1 (204.0)	302.8 (241.6)	324.3 (218.4)	306.9 (134.5)	0.70
Mean max lactate - mmol/l (SD)	8.4 (5.3)	9.4 (5.7)	8.7 (5.7)	7.2 (4.3)	0.06
Median RRT duration– hours (IQR)	83 (214)	105 (256)	84 (210)	71 (109)	0.17

*IQR* Interquartile range, *LOS* ICU length of stay, *RRT* renal replacement therapy, *MV* mechanical ventilation, *NA* noradrenaline, max: maximum. Comparisons across three age groups made using ANOVA or Kruskall-Wallis according to data distribution

### Mortality

Among the 352 patients included in the study, 171 (48.6%) died during the index hospital stay (148 in ICU, 23 on the ward). A further 62 (17.5%) died in the follow-up period (median time to death for ICU survivors 5.0 months (IQR 15.0).

**Fig. 1:**
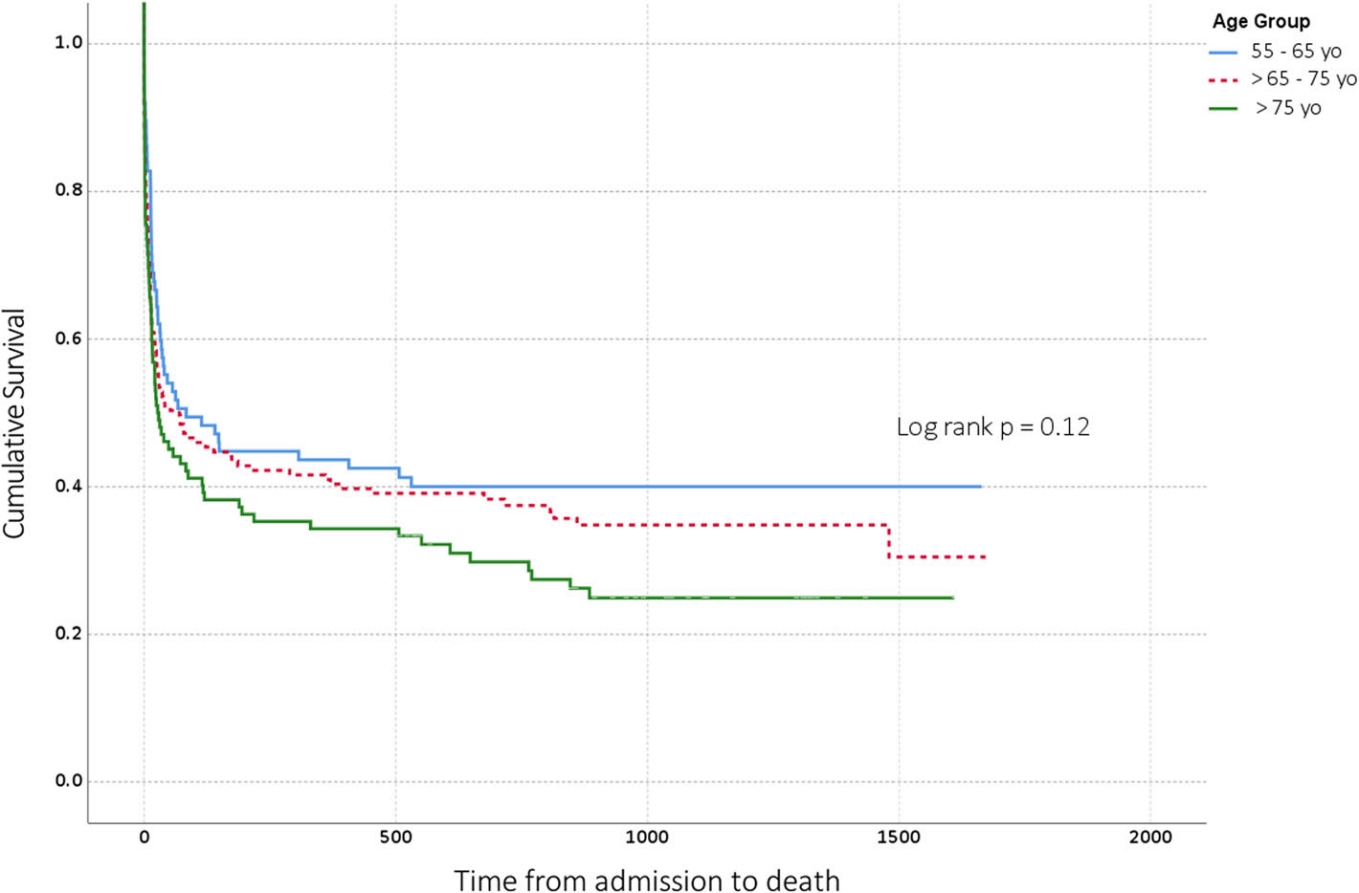
Days from admission to death. Patients are stratified according to age groups. Comparisons with log rank test. All included patients received RRT

Patients’ survival according to pre-defined age groups is displayed in Fig. 1. There was no statistically significant difference in terms of mortality between the three age groups (log rank, *p* = 0.12).

### Survey responses

One hundred nineteen patients were still alive at the time of the study and attempts were made to contact them (Fig. S2). Twenty-three could not be reached, two were unable to participate (one had language issues and one was deaf) and 11 declined participation. Hence, 83 (69.7% of survivors) agreed to respond to the survey and were included in the study. Their baseline characteristics are presented in the Supplemental material (Table S1).

### Dialysis dependence

On ICU discharge, 55 (66.3% of survey responders) were still RRT dependent, with 17 (70.8%) in G1, 28 (63.6%) in G2 and 10 (66.7%) in G3. At the time of the follow-up, six (7.2%) were still on chronic dialysis. The proportion of patients who remained RRT dependent was 0% in G1, 9% in G2 and 13.3% in G3 (*p* = 0.23).

### Quality of Life (QOL)

Overall mean VAS score was 71 (SD 22) and mean EQ-5D derived health utility 0.76 (IQR 0.26). No limitation in any of the five EQ-5D categories were reported in 39.8% of patients. Pain was the most frequently reported limitation (46.9%), followed by mobility (36.1%) and anxiety (21.6%).

**Fig. 2:**
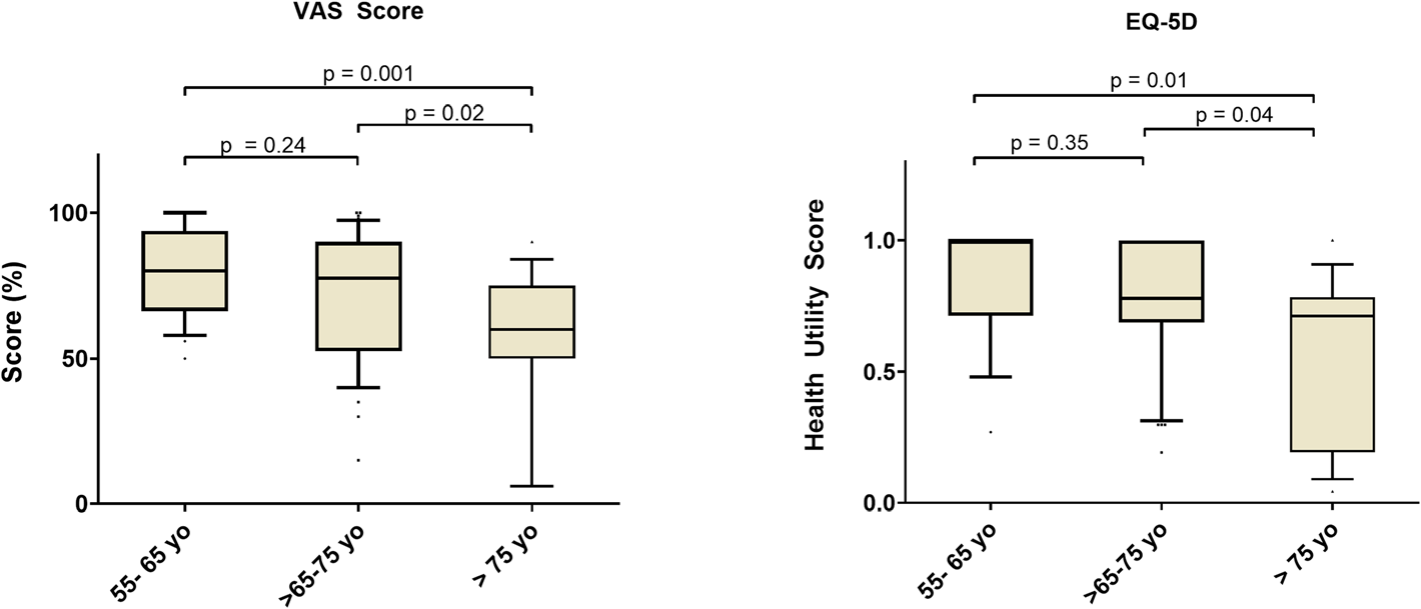
Quality of life across age groups. VAS: visual analogue scale. Data are median quartiles. Comparisons with Mann-Whitney test

**Fig. 3:**
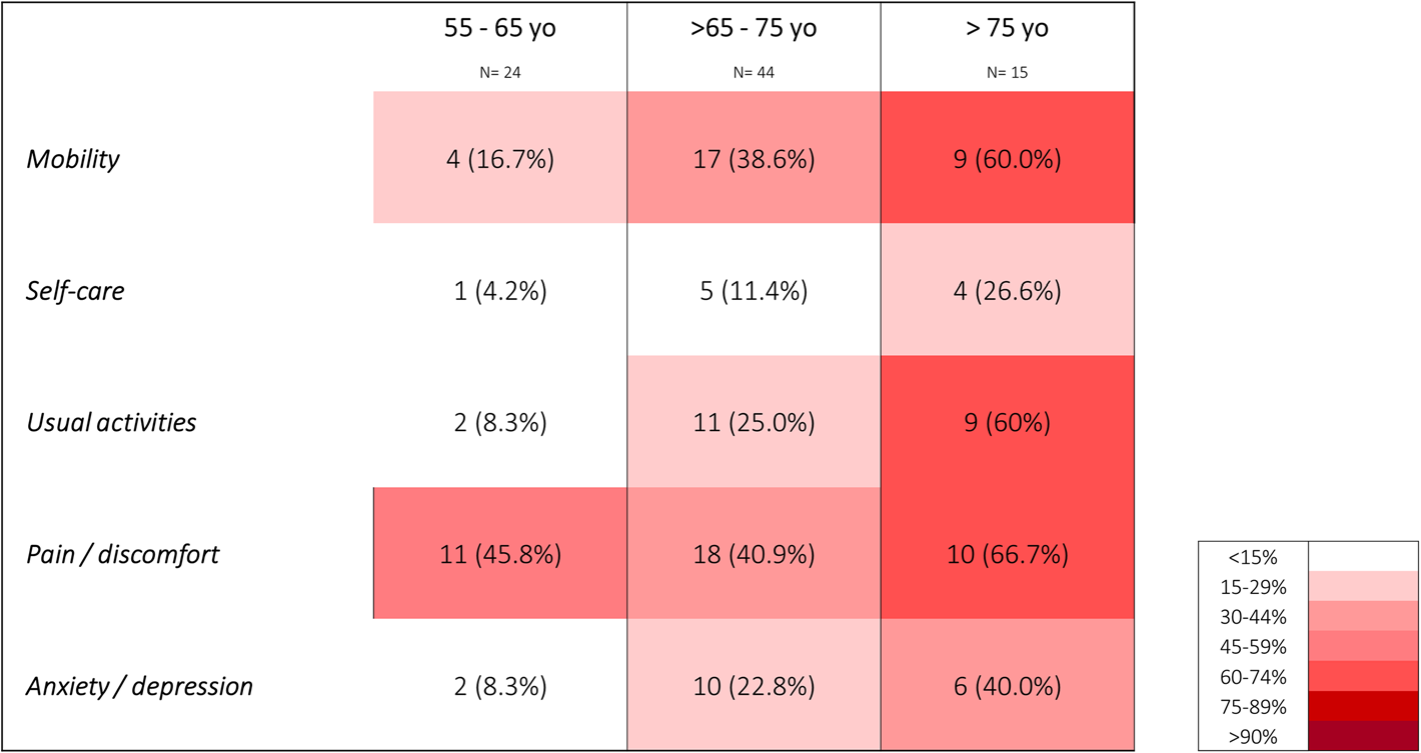
Frequency of health state limitations across age groups and EQ-5D dimensions. Heat map generated based on responses from EQ-5D survey. N (%) of patients who scored > 1 (minor or major problem) in each EQ-5D sub item

There were no significant differences in terms of QOL between patients in G1 and G2, however, they were all significantly lower in G3 patients compared to both G1 and G2 (Fig. 2). A higher proportion of patients in G3 had a VAS score of 50 or less (46.7% vs 4.2% in G1 and 25.0% in G2 *p* < 0.01). Similarly, a lower proportion of the group had no limitation at all (6.7% vs 54.2% in G1 and 43.2% in G2, *p* < 0.01). As presented in Fig. 3, the proportion of patients with limitations was higher across all five categories of the EQ-5D (mobility, self-care, usual activities, pain and anxiety). In particular, > 60% of them had limitations in either mobility, usual activities or pain.

After correction for sex, duration of hospital stay, Charlson score and SAPS on admission, age remained strongly and negatively associated with VAS (β coefficient − 1.003, 95% CI − 0.377; − 1.630) and EQ-5D derived health utility indices (β coefficient − 0.009, 95% CI: − 0.001; − 0.016).

Finally, as shown in Fig. S3, observed QOL indices were similar to predicted values in G1 and G2. Both EQ-5D and VAS were, however, lower than predicted values in patients > 75 yo (respectively 0.58 (SD 0.31) vs 0.76 (SD 0.02) [*p* value, 0.048; 95% CI − 0.002 to − 0.354] and 55 (SD 25) vs 71.7 (SD 1.9) [*p* values 0.02, 95% CI − 2.4 to − 31.0] (Table 2).

**Table 2:** Comparison of observed versus predicted (French speaking Swiss reference population)

	Observed	Predicted (1)	***p*** value	95% CI of difference	
**EQ-5D health utility**					
55–65 yo	0.84 (0.21)	0.82 (0.01)	0.74	−0.07	0.10
> 65–75 yo	0.78 (0.24)	0.79 (0.01)	0.77	−0.08	0.06
> 75 yo	0.58 (0.31)	0.76 (0.02)	0.048	−0.35	−0.002
**Visual analogue scale**					
55–65 yo	80 (15)	80.4 (1.1)	0.78	−7.26	5.50
> 65–75 yo	72 (21)	77.2 (1.2)	0.13	−11.50	1.52
> 75 yo	55 (25)	71.7 (1.9)	0.02	−31.0	−2.36

Values are mean (SD). Comparisons with one sample t-test on Observed – predicted difference

(1): Predicted values calculated according to Ref [15]

## Discussion

We performed a retrospective observational study examining long-term outcomes of patients, aged ≥55, admitted to the ICU who received RRT for AKI. We studied 352 patients over a median duration of 32.7 months. We found that, across all age groups, in-hospital and post-discharge mortality rates were very high. Compared to the rest of our cohort, older patients had a higher rate of long-term dialysis dependence and a lower QOL as assessed by EQ-5D and VAS scale. More importantly, we found that their QOL was significantly lower than an age/sex matched reference population. A higher proportion of them experienced pain, mobility limitation, depression and/or required help for their daily activities.

Long-term follow-up studies of patients undergoing RRT have reported similar mortality rates to our series (51.9%) ranging between 35 and 71% [5–9, 11, 17, 18] The even higher mortality of > 75 yo patients (73.5%) was also observed in other works: it was 61.7% in a Korean series (562 patients) and 71% in a Croatian series (178 patients) [8, 9]. This rate is not that different to the mortality observed in a group of old (75–84 yo) ICU patients who did not receive RRT (63%) [19].

Similarly, the low rate of dialysis dependence among ICU survivors is consistent with previous findings with reported rates around 5% [5, 6]. A higher (23%) rate was reported by Prskalo et al. [9], however the follow-up in this study was only 4 weeks.

Our finding that ICU survivors ≤75 yo had a similar QOL than the reference population [15], is consistent with data from Finland, where ICU survivors were found to have similar QOL compared to their baseline (pre-ICU) value, 6 months after discharge from ICU. This finding was observed in both AKI and non-AKI patients [18] as well as RRT and non-RRT patients [7]. In these two studies median age was respectively 62 and 65 yo.

However, we observed a lower than predicted QOL in > 75 yo survivors which, to the best of our knowledge, had not been described in this setting before. Most studies reporting QOL in > 80 yo ICU survivors have suggested a similar to pre-admission QOL [2]. However, among those studies very few patients had received RRT and the majority of those died.

This study has several strengths. We studied *all consecutive* patients who received RRT for AKI in our ICU during a 2.7 years period. Long-term mortality data were obtained from national statistics bureau. Hence, we are able to report reliable long-term outcomes in all patients. We managed to collect responses for the survey with a large proportion of the survivors.

However, our study also has some limitations worth discussing. First, due to our retrospective design, we were not able to record pre-admission QOL. This limitation was, in part, overcome by the ability to compare QOL with predicted values based on local reference population data. Second, this is a single center study. In particular, indications for RRT were not standardized and may not be consistent throughout the study period, in addition to being different to other centers. However, mortality rate and kidney recovery rates in our study were similar to those observed in other studies suggesting some form of external validity. Third, collecting response fora survey over the phone in geriatric patients could be seen as problematic. However, the EQ-5D has been validated for over the phone circumstances and only one patient was excluded due to deafness. Fourth, the number of patients in the > 75 yo group was small (15 patients) reflecting the very high mortality in this group. Our results should be confirmed by other studies. Fifth, the lower than predicted QOL in patients > 75 yo could have been confounded by our long-term follow-up (> 3 years in more than 60% of patients in this group). Indeed, a faster deterioration has been observed in ICU survivors compared to general population [20]. On the other hand, this long follow-up period might have selected healthier and stronger patients, because they survived a longer period and finally their QOL could be overestimated. Sixth, in the absence of a Swiss calibration, we used the French calibration to compute health utility. This appeared as a logical choice given the geographical, cultural and linguistic proximity of our region to France. Seventh, given the long term follow-up, some patients might have had multiple ICU admissions or other life events that might have altered their survival and QOL. This limitation is common to all long term follow-up studies. Finally, our study might have been underpowered to demonstrate significant difference in terms of mortality or dialysis dependence between our patient groups. Indeed, a trend for higher mortality and dialysis dependence was observed.

The observation of a lower QOL in G3 patients can appear puzzling since those patients had a lower median ICU LOS. This finding might be related to earlier withdrawal of medical care in older patients, but also demonstrates age-related limitation in post-acute illness recovery.

In summary, our study confirms that the population undergoing RRT is at very high risk of in-hospital and post-discharge death. It suggests that unlike their younger counterparts, patients > 75 yo who survive to ICU discharge have a lower QOL compared to age-matched population. This, therefore, puts in question the appropriateness of RRT initiation in such a population. Age alone cannot justify limitations of medical therapy and factors, such as frailty [21] or low pre-admission VAS score [19], should be considered before age when discussing such limitations. Age should rather correspond to a modulating factor. In addition, patients’ preference accounting for personal, cultural and educational elements must be taken into account. Our data, however, supports the view that such limitations *should be discussed* with those patients or their relatives either at the time of ICU admission or on RRT initiation.

## Conclusions

Irrespective of age, patients who received RRT for AKI have a very high in-hospital and post discharge mortality. Among survivors, the rate of RRT dependence is low. Patients > 75 yo who survived ICU had a lower QOL than younger patients. This QOL was also lower than predicted according to age and sex. The appropriateness of RRT initiation in elderly patients should be discussed according to their pre-existing QOL and frailty.

## Supplementary Information

**Additional file 1: Table S1**. Demographics of survey responders. **Figure S1**. VAS (visual analog scale). **Figure S2**. Study flow chart. **Figure S3**. Differences between observed and predicted (French speaking Swiss reference population).

## Data Availability

The datasets used and/or analyzed during the current study are available from the corresponding author upon reasonable request.

## Abbreviations

RRT: Renal Replacement Therapy
ICU: Intensive Care Unit
QoL: Quality of Life
AKI: Acute Kidney Injury
VAS: Visual Analog Scale
LOS: Length of Stay
