# Supplementary material for: Epidemiology and outcomes of elderly patients requiring renal replacement therapy in the intensive care unit: an observational study
Source: BMC Nephrol. 2021 Mar 19;22:101. doi: 10.1186/s12882-021-02302-4 (PMC7980322; doi:10.1186/s12882-021-02302-4)
Supplement: Supplementary file 1 — Additional file 1: Table S1. Demographics of survey responders. Figure S1. VAS (visual analog scale). Figure S2. Study flow chart. Figure S3. Differences between observed and predicted (French speaking Swiss reference population). [file 12882_2021_2302_MOESM1_ESM.docx]

# epidemiology and outcome of elderly patients after renal replacement therapy in the intensive care unit: an observational study: Supplementary material

**Figure S1: VAS (visual analog scale)** French version, as provided by EuroQol for the use over the phone. This version has been sent to the survivors prior to the phone call.

Excluded:

- < 55 yo (135)
- Chronic dialysis (84)
- Declined data reutilization (37)

Died in ICU (148)

Died on the ward (23)

Could not be reached (23)

Unable to participate (2)

Declined participation (11)

Died during follow-up period (62)

**Figure S2: Study flow chart.**


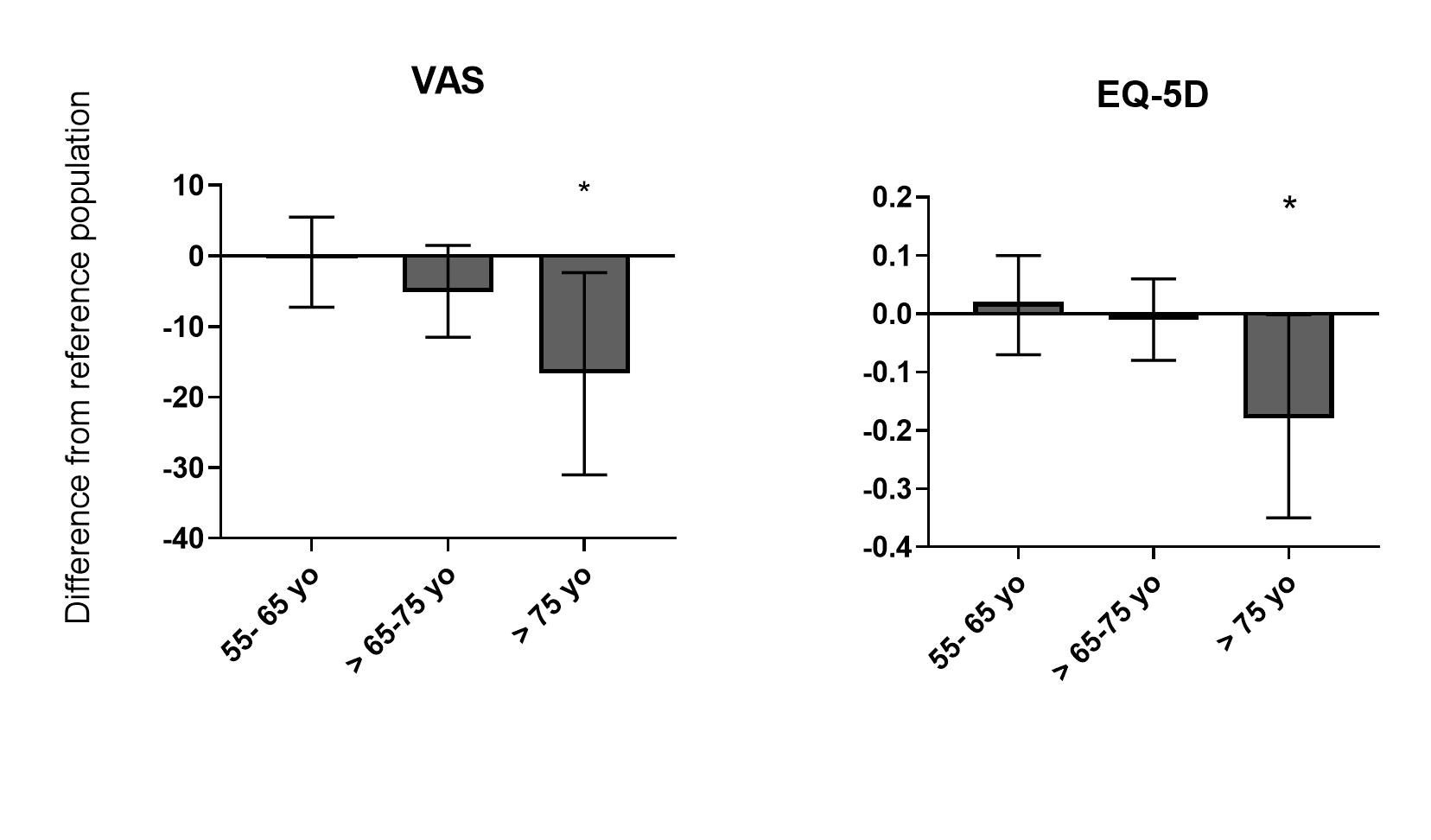


**Figure S3: Differences between observed and predicted (French speaking Swiss reference population).**

Values are mean (SD). Comparisons with one sample t-test on Observed – predicted difference.

Predicted values calculated according to Ref 15 (Perneger et al).

|  | **TOTAL** | **55-65 yo** | **>65 – 75 yo** | **>75 yo** | **p value** |
| --- | --- | --- | --- | --- | --- |
| N (%) | 83 (100%) | 24 (28.9%) | 44 (53.0%) | 15 (18.1%) |  |
| Median age - years (IQR) | 68.1 (8.6) | 59.9 (5.9) | 68.9 (3.6) | 78.6 (5.0) | <0.001 |
| Gender Male - n (%) | 60 (72.3%) | 21 (87.5%) | 28 (63.6%) | 11 (73.3%) | 0.11 |
| Median weight – kg (IQR) | 85 (28) | 87 (26) | 85.5 (29.0) | 85 (23) | 0.72 |
| Admission year |  |  |  |  | 0.18 |
| 2015 | 21 (25.3%) | 7 (29.2%) | 12 (27.3%) | 2 (13.3%) |  |
| 2016 | 26 (31.3%) | 6 (25.0%) | 13 (29.5%) | 7 (46.7%) |  |
| 2017 | 26 (31.3%) | 10 (41.7%) | 14 (31.8%) | 2 (13.3%) |  |
| 2018 | 10 (12.0%) | 1 (4.2%) | 5 (11.4%) | 4 (26.7%) |  |
| Admission type |  |  |  |  |  |
| Medical | 36 (43.4%) | 9 (37.5%) | 24 (54.5%) | 3 (20.0%) | 0.08 |
| Surgical | 43 (51.8%) | 15 (62.5%) | 17 (38.6%) | 11 (73.3%) |  |
| Other | 4 (4.8%) | 0 (0%) | 3 (6.8%) | 1 (6.7%) |  |
| Median SAPS Score (IQR) |  | 53 (22) | 56 (13) | 59 (13) | 0.27 |
| Median ICU LOS – days (IQR) | 10.2 (17.6) | 9.8 (14.0) | 10.6 (22.3) | 6.9 (17.1) | 0.99 |
| Median hospital LOS – days (IQR) | 38.9 (50.7) | 34.3 (51.2) | 34.0 (50.1) | 40.9 (42.2) | 0.57 |
| Co-existing conditions – n (%) | 38 (45.8%) | 12 (50.0%) | 19 (43.2%) |  | 0.86 |
| CKD | 21 (25.9%) | 5 (21.7%) | 15 (34.9%) | 1 (6.7%) | 0.09 |
| CHD | 14 (16.9%) | 5 (21.7%) | 7 (15.9%) | 2 (13.3%) | 0.81 |
| Chron.resp.D | 14 (16.9%) | 1 (4.2%) | 8 (18.2%) | 5 (33.3%) | 0.06 |
| Chron.liver.D | 7 (8.4%) | 4 (16.7%) | 3 (6.8%) | 0 | 0.16 |
| Immunosupp. | 8 (9.6%) | 4 (16.7%) | 4 (9.1%) | 0 | 0.23 |
| Haematological | 3 (3.6%) | 2 (8.3%) | 1 (2.3%) | 0 | 0.31 |
| Metastatic cancer | 0 (0%) | 0 (0%) | 0 (0%) | 0 (0%) | 1 |
| On RRT initiation |  |  |  |  |  |
| MV on initiation | 56 (67.5%) | 18 (75.0%) | 27 (61.4%) | 11 (73.3%) | 0.45 |
| NA on initiation | 68 (81.9%) | 20 (83.3%) | 33 (75.0%) | 15 (100%) | 0.23 |
| Mean max creatinine - mmol/l (SD) | 366 (254) | 309 (207) | 419 (300) | 298 (106) | 0.16 |
| Mean max lactate - mmol/l (SD) | 6.2 (2.9) | 6.2 (2.0) | 5.8 (2.7) | 6.8 (4.3) | 0.72 |

**Table S1: Demographics of survey responders**

*IQR: Interquartile range, LOS*: ICU length of stay, *RRT*: renal replacement therapy, *MV*: mechanical ventilation, *NA*: noradrenaline, *max*: maximum.
